# Effects of *Lindera aggregata* Linderae Radix on Testosterone Propionate-Induced Benign Prostatic Hyperplasia in Rats

**DOI:** 10.3390/plants15203203

**Published:** 2026-10-19

**Authors:** Sehwang Kang, Sueyoung An, Mi-Hyang Yoo, Enmei Gao, Jungbin Song

**Affiliations:** 1 Graduate School, Kyung Hee University, 26 Kyungheedae-ro, Dongdaemun-gu, Seoul 02447, Republic of Korea; stork_river@naver.com (S.K.); asy9626@gmail.com (S.A.); 2 Department of Herbal Pharmacology, College of Korean Medicine, Kyung Hee University, 26 Kyungheedae-ro, Dongdaemun-gu, Seoul 02447, Republic of Korea; ymih1991@khu.ac.kr; 3 Song-Ahn Institute for Natural Therapeutics (SAINT), ILMOK Bio Inc., 26 Kyungheedae-ro, Dongdaemun-gu, Seoul 02447, Republic of Korea; emgao@ilmokbio.com

**Keywords:** *Lindera aggregata*, Linderae Radix, benign prostatic hyperplasia, testosterone, dihydrotestosterone, apoptosis

## Abstract

Linderae Radix, the root of *Lindera aggregata*, has shown beneficial effects on bladder function in experimental studies, but whether it can attenuate benign prostatic hyperplasia (BPH) remains unknown. This study evaluated an ethanol extract of *L. aggregata* roots (LAE) in a testosterone propionate (TP)-induced rat model of prostatic hyperplasia. Castrated male Sprague–Dawley rats received TP (3 mg/kg/day, subcutaneously) and LAE (200 mg/kg/day) or finasteride (1 mg/kg/day) orally for 14 days. LAE significantly reduced total and ventral prostate weights and indices, together with ventral prostate histological scores and epithelial thickness. The serum dihydrotestosterone/testosterone ratio was significantly lower in the LAE group than in the control group. In the ventral prostate, aromatase protein expression was significantly reduced by LAE treatment. Significant changes in apoptosis-related proteins included increased Bax and cleaved caspase-3 levels, decreased procaspase-9 levels, and higher cleaved/procaspase ratios of both caspases. These findings demonstrate that LAE attenuated TP-induced prostatic hyperplasia in rats. The accompanying hormonal and protein expression changes provide a basis for further mechanistic investigation. Further evaluation of LAE as a potential therapeutic option for BPH is warranted.

## 1. Introduction

Benign prostatic hyperplasia (BPH) is defined histologically by the proliferation of smooth muscle and epithelial cells in the prostate [1]. Prostatic growth continues beyond puberty, and age-related hyperplasia predominantly develops in the transition zone surrounding the urethra [2]. Prostatic enlargement may compress the urethra and contribute to lower urinary tract symptoms (LUTS), including storage symptoms such as frequency, nocturia, and urgency and voiding symptoms such as a weak or intermittent stream, straining, and hesitancy [3]. BPH is highly prevalent among older men, and the estimated global number of prevalent cases increased from approximately 50.7 million in 1990 to 112.5 million in 2021 [4].

BPH has a multifactorial pathophysiology involving age-related hormonal alterations, genetic predisposition, growth factor signaling, chronic inflammation, and metabolic factors [5,6]. In particular, androgen imbalance plays an important role in prostatic growth and BPH development [2]. Testosterone and dihydrotestosterone (DHT) are the principal androgens implicated in disease development and progression. DHT, produced from testosterone by 5α-reductase, is a potent endogenous androgen because it binds to the androgen receptor with higher affinity and dissociates more slowly than testosterone [7]. Higher serum DHT concentrations and DHT/total testosterone ratios have been associated with larger prostate volume and a higher prevalence of BPH [8], with prospective evidence also linking higher serum DHT concentrations to subsequent BPH risk [9]. These observations support androgen metabolism as a relevant therapeutic target in BPH.

Current pharmacological management of BPH includes α_1_-blockers and 5α-reductase inhibitors (5-ARIs), used alone or in combination [10]. α_1_-Blockers relieve LUTS by relaxing smooth muscle in the prostate and bladder neck, but they do not reduce prostate size and are associated with ejaculatory dysfunction [11]. In contrast, 5-ARIs reduce prostate size but can cause sexual adverse effects, including erectile dysfunction and decreased libido [11,12]. These limitations have contributed to continued interest in herbal medicines as complementary approaches for BPH management [13].

Linderae Radix, the root of *Lindera aggregata* (Sims) Kosterm., is a traditional East Asian herbal medicine with records dating to the eighth century [14]. It has traditionally been used for chest and abdominal pain, dysmenorrhea, enuresis, and urinary frequency [15]. Linderae Radix contains sesquiterpenoids, such as linderane and linderalactone, and alkaloids, such as norisoboldine, boldine, and isoboldine [16]. Preclinical studies have reported anti-inflammatory [17], analgesic [18], anti-arthritic [19], and anticancer [20] activities of Linderae Radix extracts and their constituents.

Urinary frequency, one of the traditional indications for Linderae Radix [15], can occur in a range of urological and systemic conditions, including BPH, overactive bladder, bladder outlet obstruction, and diabetes mellitus [3,21]. Linderae Radix extract improved detrusor contractility and attenuated bladder remodeling in mice with late-stage diabetic bladder dysfunction [22]. A three-herb formula containing Linderae Radix also improved bladder function in aging female rats and ameliorated bladder overactivity in diabetic rats [23,24]. These findings suggest beneficial effects of Linderae Radix on bladder function; however, it remains unclear whether Linderae Radix can suppress BPH. Therefore, this study evaluated the effects of *L. aggregata* root extract (LAE) and explored its potential mechanisms in a testosterone propionate (TP)-induced BPH rat model.

## 2. Results

### 2.1. High-Performance Liquid Chromatography (HPLC) Analysis of LAE

The norisoboldine peak matched the reference standard in retention time and UV spectrum, with no apparent interference. The analytical method for norisoboldine showed good linearity over 200–1000 µg/mL (R^2^ = 0.9998). The limit of detection (LOD) and limit of quantification (LOQ) were 8.526 and 25.536 µg/mL, respectively. Accuracy assessed using independently prepared standard solutions ranged from 100.8% to 103.9%. The relative standard deviation (RSD) was 0.92% for repeatability and 3.76% for intermediate precision.

**Figure 1:**
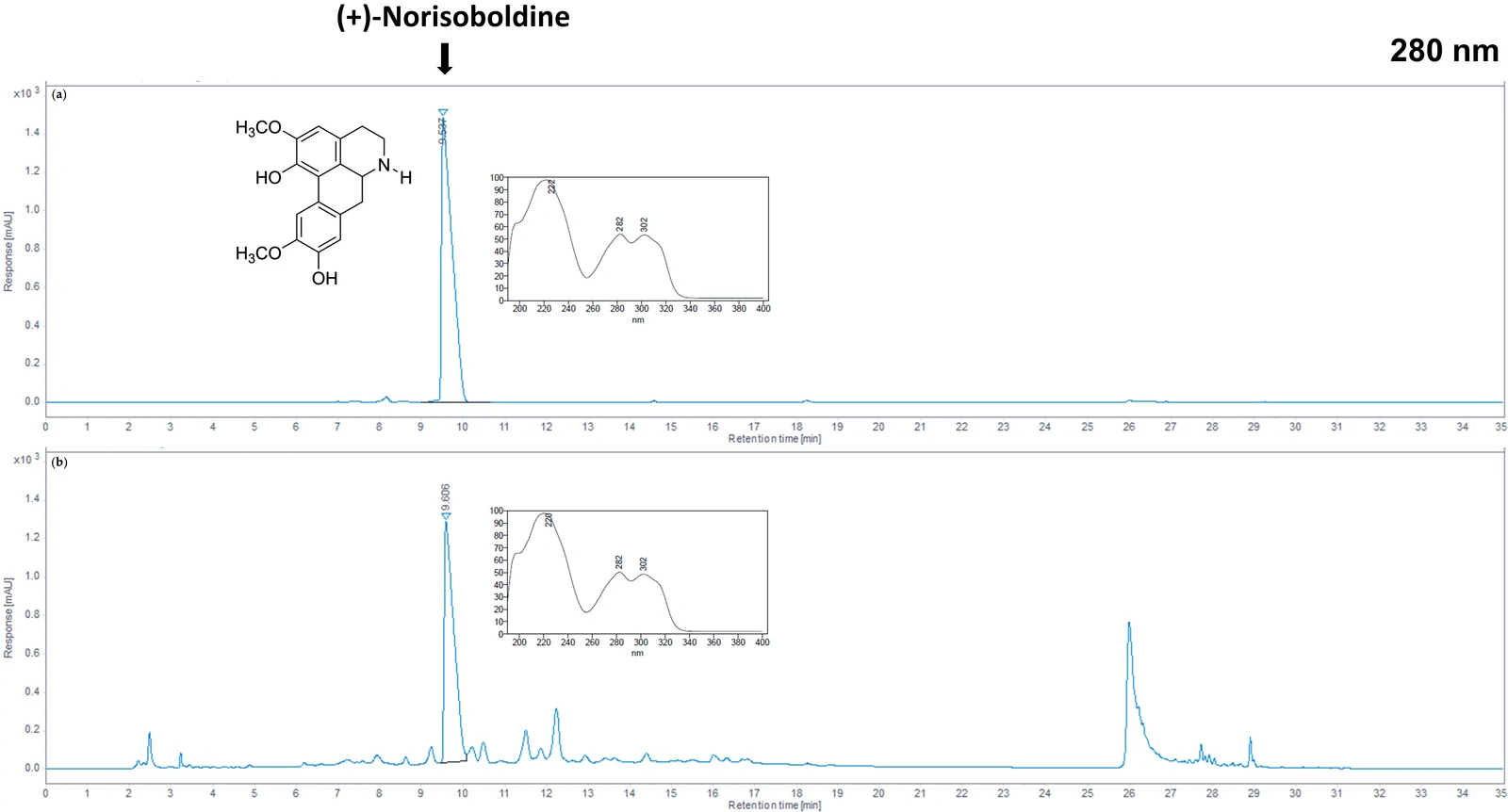
Representative high-performance liquid chromatography chromatograms of (**a**) norisoboldine reference standard and (**b**) *Lindera aggregata* root extract (LAE), detected at 280 nm. Norisoboldine was identified by comparison of its retention time and UV absorption spectrum with those of the authentic reference standard. The chemical structure of (+)-norisoboldine is shown in panel (**a**).

The norisoboldine peak in LAE was detected at a retention time of approximately 9.6 min (Figure 1). Quantitative analysis showed that LAE contained 52.2 mg of norisoboldine per gram of extract.

### 2.2. Prostate Weight and Prostate Index

**Figure 2:**
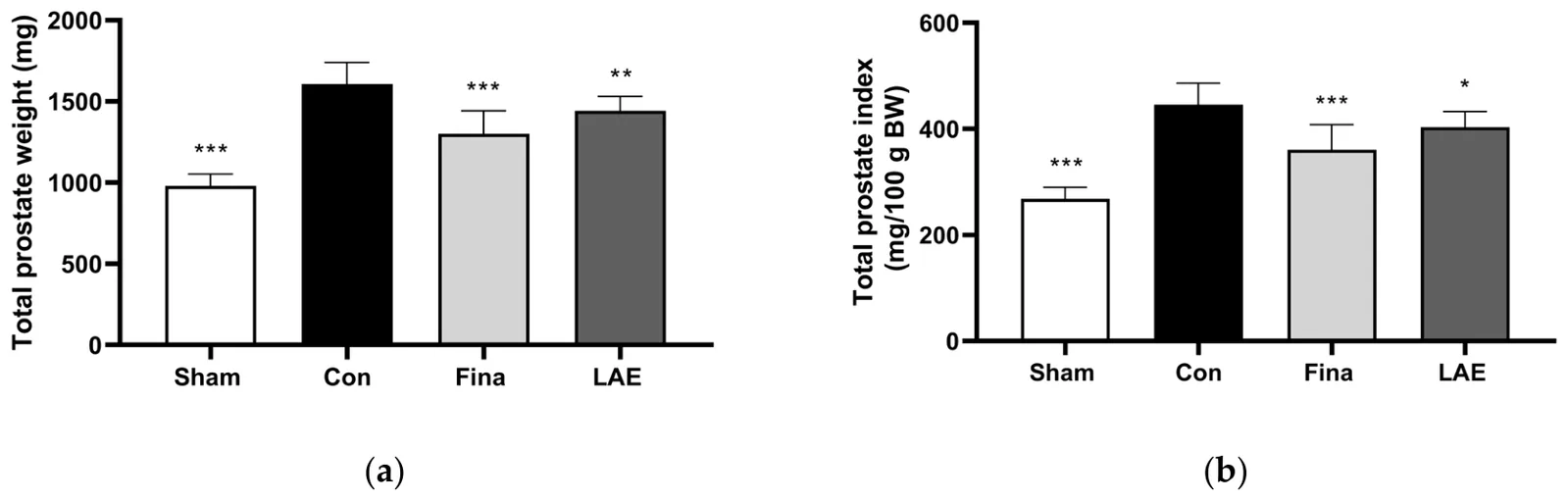

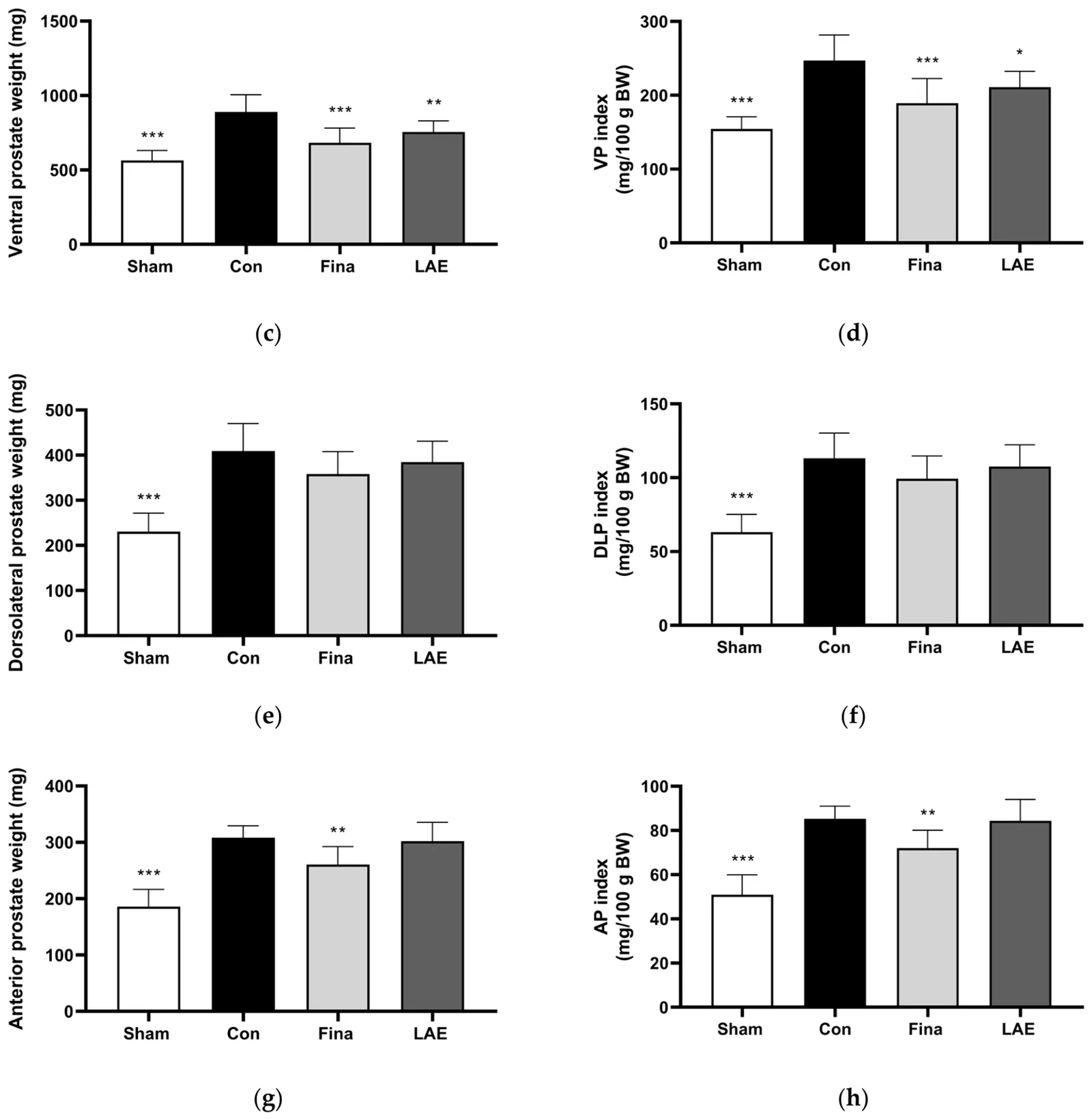
Effects of *Lindera aggregata* root extract (LAE) on prostate weight and prostate index in a testosterone propionate-induced benign prostatic hyperplasia rat model. (**a**) Total prostate weight; (**b**) total prostate index; (**c**) ventral prostate (VP) weight; (**d**) VP index; (**e**) dorsolateral prostate (DLP) weight; (**f**) DLP index; (**g**) anterior prostate (AP) weight; (**h**) AP index. * *p* < 0.05, ** *p* < 0.01, and *** *p* < 0.001 vs. control (Con). BW, body weight; Fina, finasteride 1 mg/kg; LAE, *L. aggregata* root extract 200 mg/kg.

Two weeks of TP administration significantly increased the total prostate weight and prostate index in the control group compared with the sham group (both *p* < 0.001; Figure 2a,b). LAE at 200 mg/kg significantly reduced the total prostate weight from 1608.1 ± 132.7 mg in the control group to 1442.1 ± 88.6 mg (*p* < 0.01) and the prostate index from 445.6 ± 40.8 to 403.2 ± 29.8 (*p* < 0.05), corresponding to 26.5% and 24.0% inhibition of the TP-induced increases, respectively. For the total prostate index, the standardized mean difference between the LAE and control groups was −1.16 (Hedges’ *g*; 95% CI [−2.00, −0.30]).

Lobe-specific analysis showed that LAE significantly reduced both the weight and index of the ventral prostate compared with the control group (Figure 2c,d). In contrast, LAE did not significantly affect the weight or index of the dorsolateral or anterior prostate (Figure 2e–h). No adverse events were observed throughout the study.

### 2.3. Histopathological Findings and Morphometric Analysis of the Ventral Prostate

#### 2.3.1. Histopathological Assessment by Hematoxylin and Eosin (H&E) Staining

**Figure 3:**
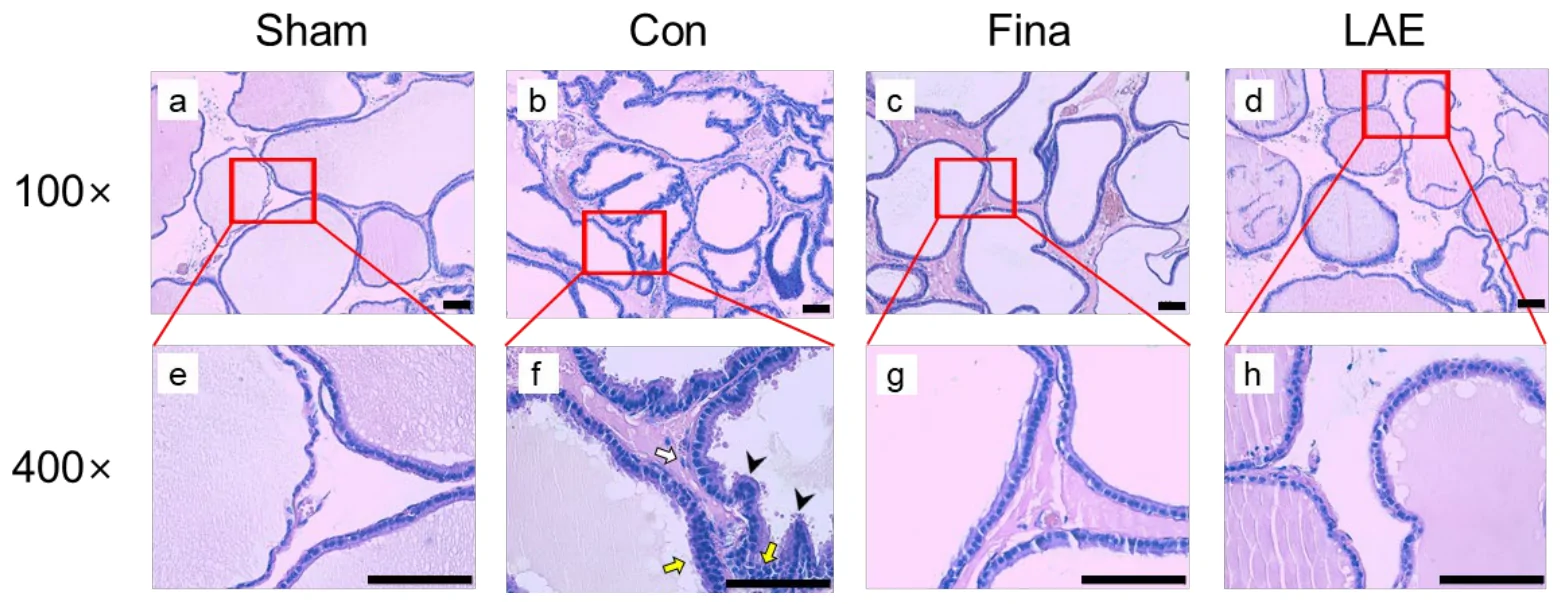
Representative hematoxylin and eosin (H&E)-stained sections of the rat ventral prostate. Images are shown for the sham (**a**,**e**), control (**b**,**f**), finasteride-treated (1 mg/kg; (**c**,**g**)), and *Lindera aggregata* root extract (LAE)-treated (200 mg/kg; (**d**,**h**)) groups. Upper panels (**a**–**d**) show 100× magnification, and lower panels (**e**–**h**) show the corresponding boxed regions at 400× magnification. Black arrowheads indicate papillary or villous projections, yellow arrows indicate epithelial stratification, and white arrows indicate increased stromal cellularity. Con, control; Fina, finasteride 1 mg/kg. Scale bars: 100 μm.

H&E staining of the ventral prostate showed intact glandular morphology in the sham group, with a uniform epithelial lining and normal stromal appearance (Figure 3a,e). TP treatment produced marked histopathological abnormalities in the control group, including epithelial thickening and stratification, papillary or villous projections into the lumen, and increased stromal cellularity (Figure 3b,f). In rats treated with LAE at 200 mg/kg, these abnormalities were less prominent, with reduced epithelial thickening, fewer luminal projections, and a more regular glandular architecture than in the control group (Figure 3d,h). Finasteride treatment similarly ameliorated the histological abnormalities (Figure 3c,g).

#### 2.3.2. Histological Scoring and Epithelial Thickness Analysis

Quantitative histological assessment showed a higher total histological score in the control group than in the sham group (Table 1). LAE treatment at 200 mg/kg significantly reduced the total histological score from 53.60 ± 10.84 in the control group to 27.26 ± 8.92 (*p* < 0.001), approaching the value observed in the sham group (25.40 ± 3.99). LAE significantly reduced the scores for acinar shape, the number and distribution of epithelial layers, polarity, epithelial piling, isolated epithelial clusters, lesion distribution, mitotic figures, and basement membrane thickness compared with the control group. Detailed scores for individual histopathological parameters are presented in Table 1.

**Figure 4:**
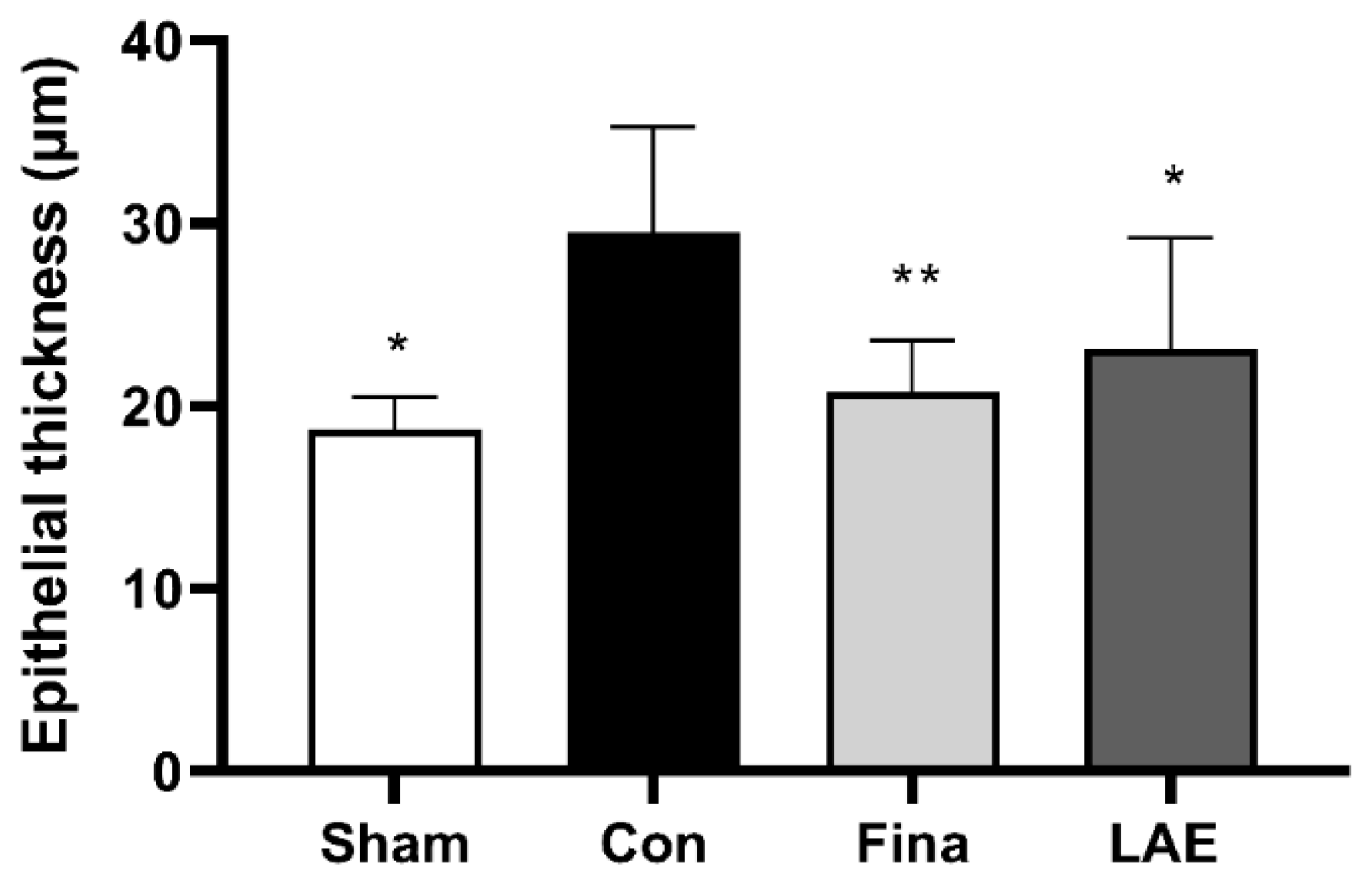
Effects of *Lindera aggregata* root extract on epithelial thickness in the ventral prostate. * *p* < 0.05 and ** *p* < 0.01 vs. control (Con). Fina, finasteride 1 mg/kg; LAE, *L. aggregata* root extract 200 mg/kg.

Morphometric analysis showed that LAE treatment at 200 mg/kg significantly reduced epithelial thickness compared with the control group (23.1 ± 6.1 vs. 29.5 ± 5.8 μm, *p* < 0.05; Figure 4).

### 2.4. Serum Testosterone and DHT Levels

**Figure 5:**
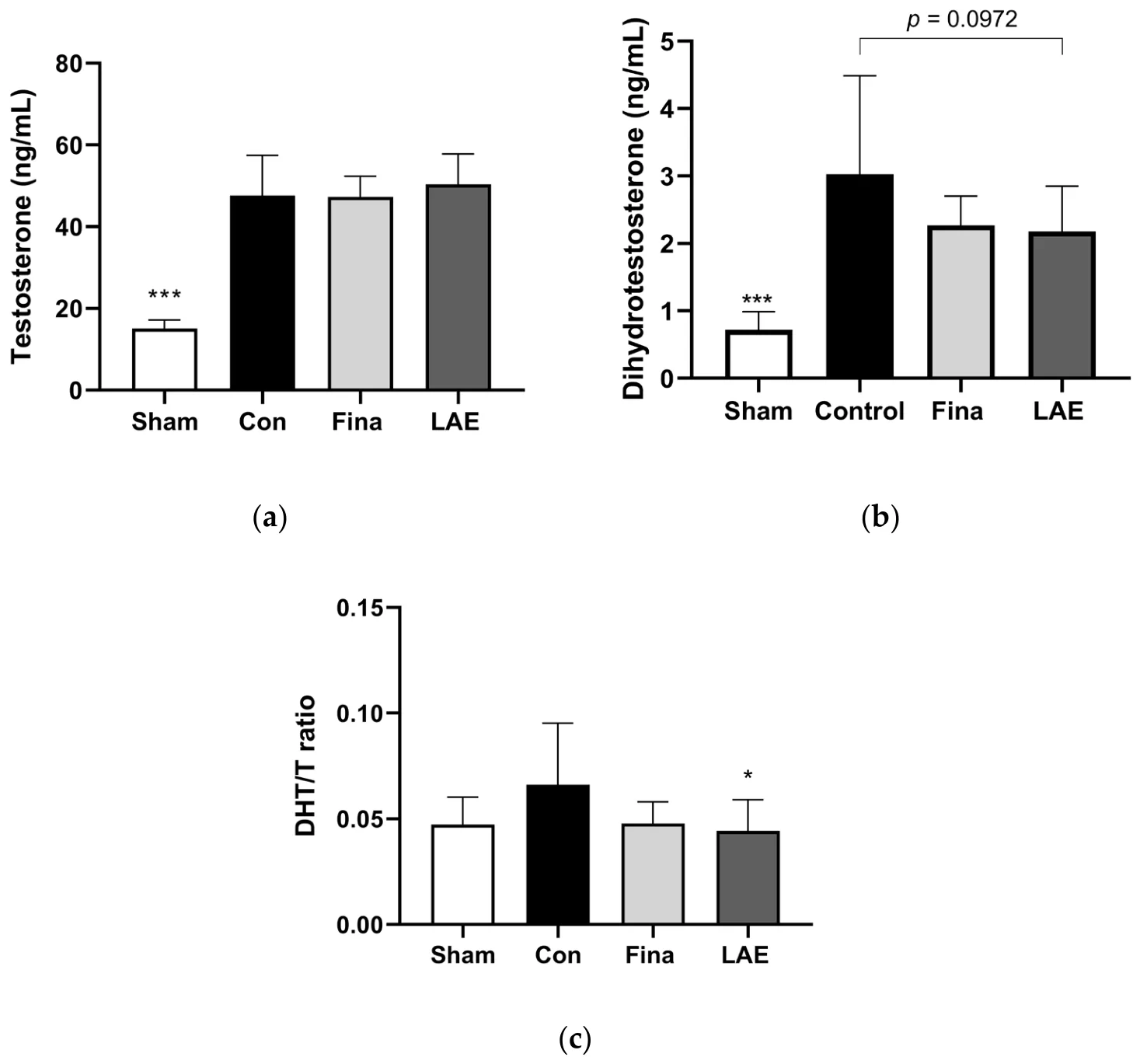
Effects of *Lindera aggregata* root extract on serum (**a**) testosterone, (**b**) dihydrotestosterone (DHT), and (**c**) DHT/testosterone (DHT/T) ratio. * *p* < 0.05 and *** *p* < 0.001 vs. control (Con). Fina, finasteride 1 mg/kg; LAE, *Lindera aggregata* root extract 200 mg/kg.

TP administration significantly increased serum testosterone and DHT levels in the control group compared with the sham group (both *p* < 0.001; Figure 5a,b). Serum testosterone levels did not differ significantly among the TP-treated groups. LAE treatment showed a trend toward lower serum DHT levels compared with the control group (2.2 ± 0.7 vs. 3.0 ± 1.5 ng/mL, *p* = 0.0972; Figure 5b), while significantly reducing the DHT/testosterone (DHT/T) ratio (*p* < 0.05; Figure 5c).

### 2.5. 5α-Reductase Type 2 and Aromatase Expression in the Ventral Prostate

**Figure 6:**
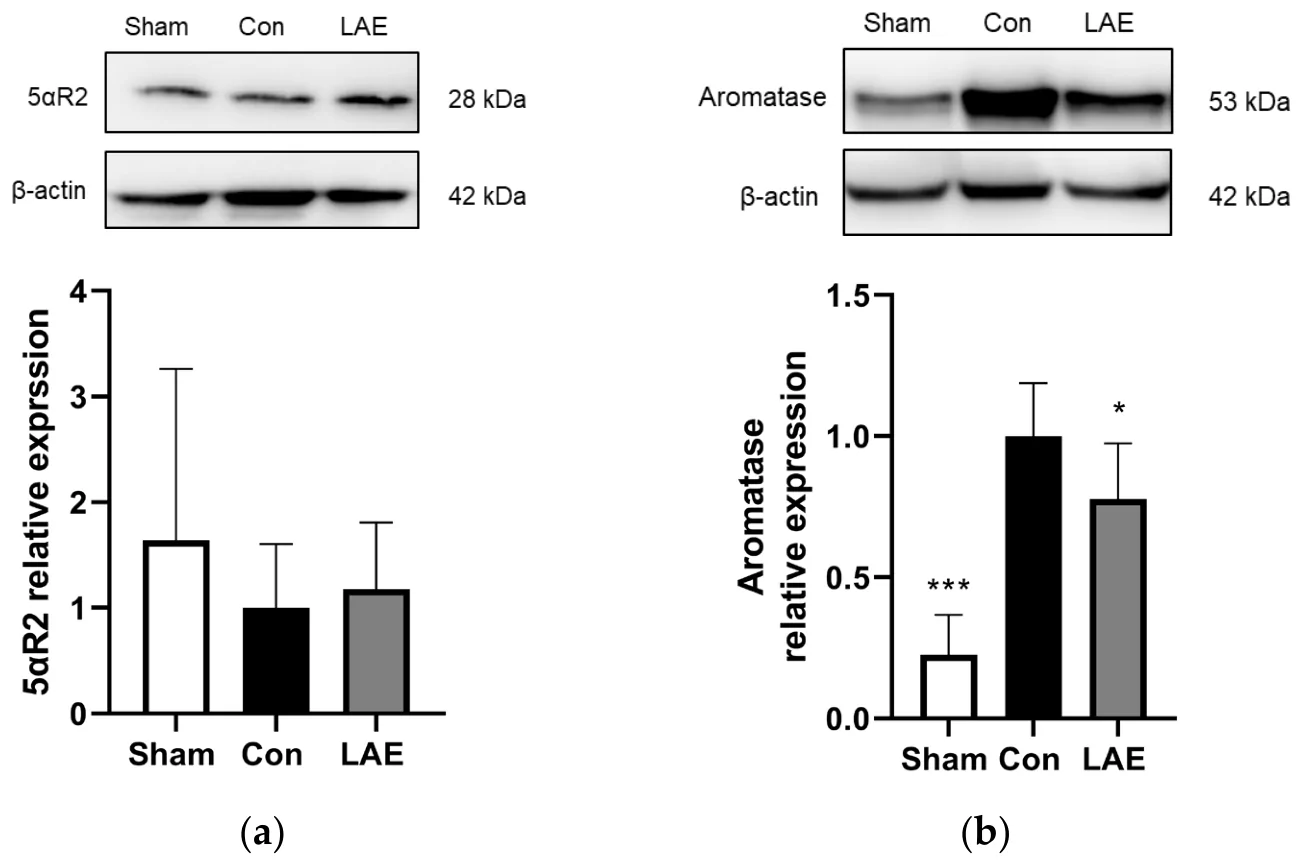
Effects of *Lindera aggregata* root extract (LAE) on 5α-reductase type 2 and aromatase protein expression in the ventral prostate. Representative Western blot images and quantitative analyses of (**a**) 5α-reductase type 2 and (**b**) aromatase expression. β-Actin was used as the loading control. * *p* < 0.05 and *** *p* < 0.001 vs. control (Con). LAE, *L. aggregata* root extract 200 mg/kg.

Western blot analysis of ventral prostate tissue revealed no significant difference in 5α-reductase type 2 protein expression between the LAE and control groups (Figure 6a). LAE at 200 mg/kg significantly reduced aromatase protein expression compared with the control group (Figure 6b).

### 2.6. Apoptosis-Related Protein Expression in the Ventral Prostate

#### 2.6.1. Bcl-2 and Bax Expression

**Figure 7:**
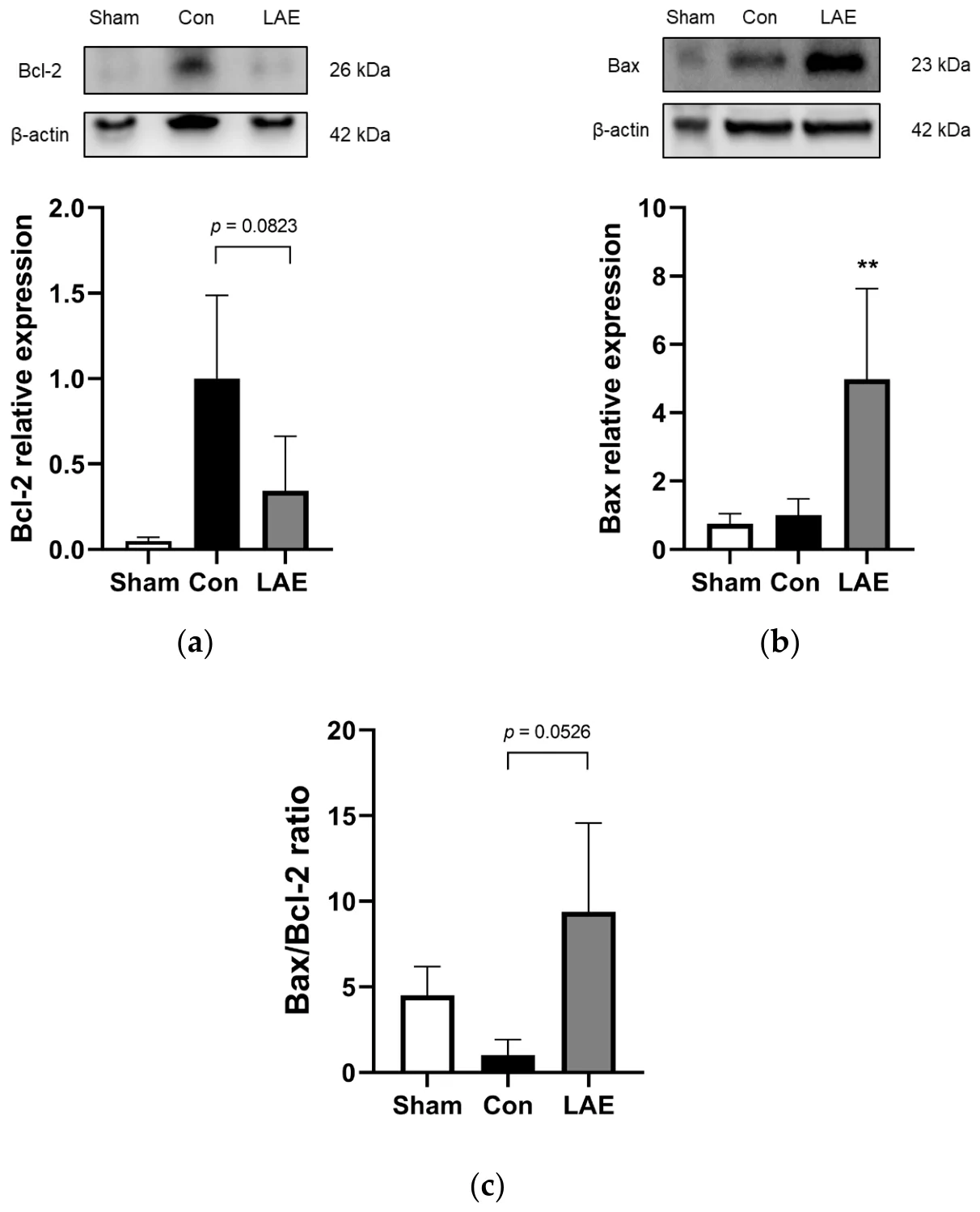
Effects of *Lindera aggregata* root extract (LAE) on Bcl-2 and Bax protein expression in the ventral prostate. Representative Western blot images and quantitative analyses of (**a**) Bcl-2 and (**b**) Bax expression, and (**c**) Bax/Bcl-2 ratio calculated from the quantified protein expression levels. β-Actin was used as the loading control. ** *p* < 0.01 vs. control (Con). LAE, *L. aggregata* root extract 200 mg/kg.

Western blot analysis showed that Bcl-2 expression was lower in the LAE-treated group than in the control group, although the difference was not statistically significant (*p* = 0.0823; Figure 7a). In contrast, LAE treatment at 200 mg/kg significantly increased Bax expression compared with the control group (*p* < 0.01; Figure 7b). The Bax/Bcl-2 ratio showed an increasing trend following LAE treatment (*p* = 0.0526; Figure 7c).

#### 2.6.2. Caspase-9 and Caspase-3 Expression

**Figure 8:**
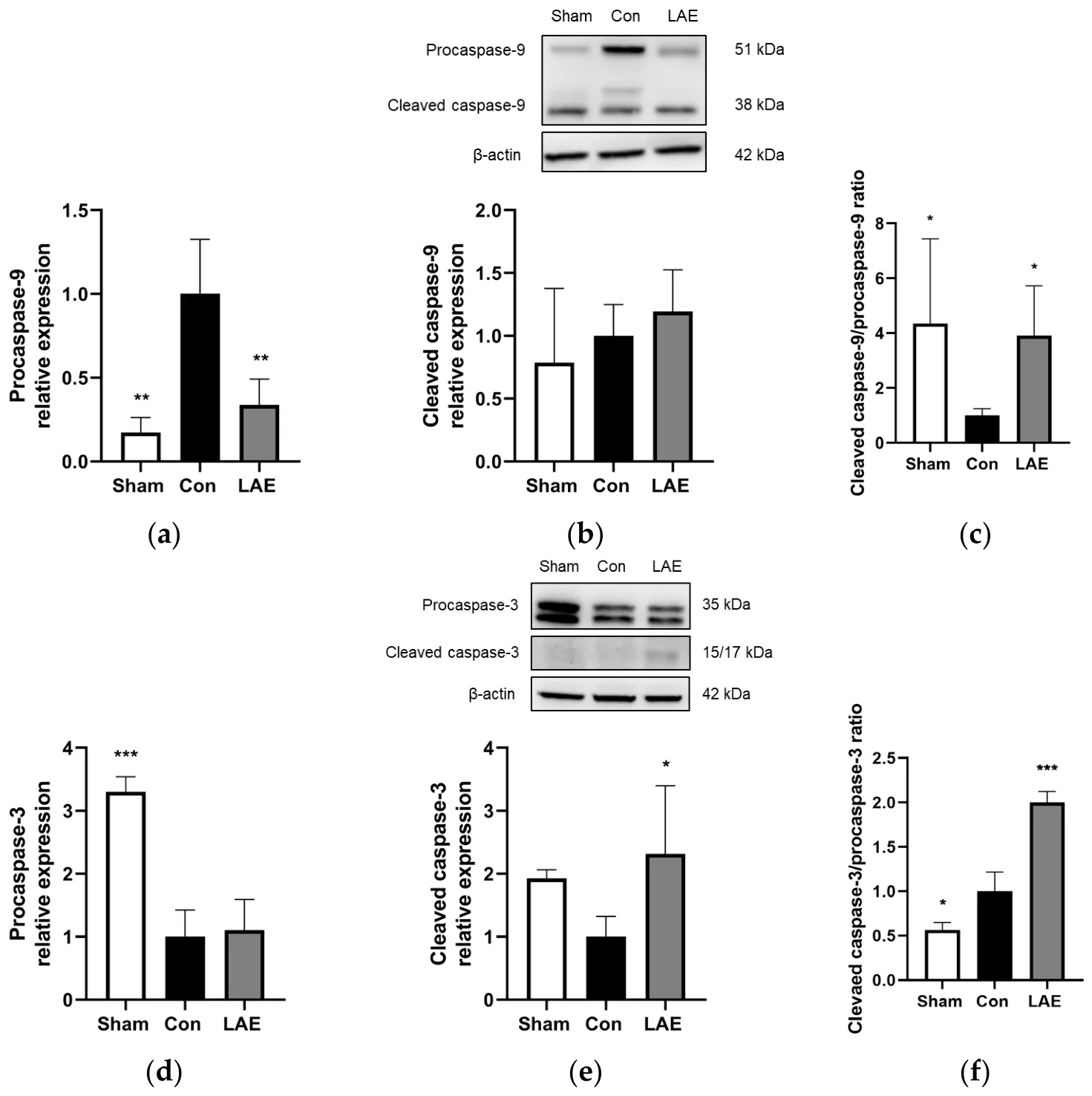
Effects of *Lindera aggregata* root extract on caspase-9 and caspase-3 protein expression in the ventral prostate. Representative Western blot images are shown above the corresponding quantitative analyses of (**a**) procaspase-9, (**b**) cleaved caspase-9, (**c**) cleaved caspase-9/procaspase-9 ratio, (**d**) procaspase-3, (**e**) cleaved caspase-3, and (**f**) cleaved caspase-3/procaspase-3 ratio. β-Actin was used as the loading control. * *p* < 0.05, ** *p* < 0.01, and *** *p* < 0.001 vs. control (Con). LAE, *L. aggregata* root extract 200 mg/kg.

LAE treatment at 200 mg/kg significantly decreased procaspase-9 expression (*p* < 0.01; Figure 8a), while cleaved caspase-9 expression remained unchanged (Figure 8b). The cleaved caspase-9/procaspase-9 ratio was significantly increased following LAE treatment (*p* < 0.05; Figure 8c).

Procaspase-3 expression did not differ significantly between the LAE-treated and control groups (Figure 8d). In contrast, LAE significantly increased cleaved caspase-3 expression (*p* < 0.05; Figure 8e) and the cleaved caspase-3/procaspase-3 ratio (*p* < 0.01; Figure 8f).

## 3. Discussion

Although Linderae Radix has traditionally been used for LUTS and previous experimental studies have demonstrated beneficial effects on bladder function [15,22,23,24], its effects on prostatic hyperplasia have not been investigated. In the present study, LAE attenuated TP-induced prostatic hyperplasia, as demonstrated by reductions in prostate enlargement and improvements in histopathological changes. To our knowledge, this is the first experimental evidence demonstrating an inhibitory effect of Linderae Radix as a single-herb extract on androgen-induced prostatic hyperplasia.

Following 2 weeks of TP administration, LAE at 200 mg/kg/day significantly reduced the total and ventral prostate indices compared with the control group. The castrated TP-induced rat model provides a reproducible system for evaluating androgen-dependent prostatic growth, as orchiectomy minimizes variability in endogenous androgen production and repeated TP administration induces prostate enlargement and epithelial hyperplasia within a relatively short period [25]. Spontaneous BPH models in dogs and chimpanzees better reflect the age-dependent features of human BPH, but practical constraints limit their routine use in pharmacological studies [26,27]. Given the important role of androgen-dependent signaling in BPH development [2], the TP-induced rat model is widely used to evaluate agents targeting androgen-driven prostatic growth [25]. In the present study, the inhibitory effect of LAE was particularly evident in the ventral prostate. The rat ventral prostate is highly androgen responsive and exhibits distinct androgen-dependent gene regulation compared with other prostatic lobes [28]. Thus, the reduction in ventral prostate enlargement supports an inhibitory effect of LAE on androgen-stimulated prostatic growth.

The LAE dose of 200 mg/kg/day was selected with reference to previous in vivo studies demonstrating the pharmacological activity of ethanolic extracts of Linderae Radix [29,30]. Based on the extraction yield of 12.05%, this dose corresponds to approximately 1.66 g/kg/day of crude Linderae Radix, which is close to the 2 g/kg dose at which a 75% ethanol extract showed hepatoprotective effects in rats [30]. Using body surface area-based dose conversion, the LAE dose corresponds to a human equivalent dose of approximately 1.95 g/day for a 60-kg adult [31], equivalent to approximately 16 g/day of crude Linderae Radix.

The reductions in prostate weight and index were accompanied by improvements in glandular architecture, providing histological support for the inhibitory effect of LAE on TP-induced prostatic hyperplasia. Epithelial thickening and intraluminal projections have been reported in a testosterone-induced rat model of prostatic hyperplasia [32]. In the present study, LAE reduced epithelial thickness and the total histological score, with improvements in scores for epithelial layering, piling, and polarity, suggesting attenuation of epithelial disorganization in the ventral prostate.

LAE treatment showed a trend toward lower serum DHT levels and a significant decrease in the DHT/T ratio, whereas testosterone levels did not differ significantly among the TP-treated groups. The importance of DHT formation in BPH is supported by clinical evidence that finasteride, a 5α-reductase inhibitor, reduces the risk of disease progression [33]. Observational studies have associated higher serum DHT/T ratios with larger prostate volume [8] and higher reciprocal T/DHT ratios with a lower subsequent risk of BPH [9]. Higher DHT/T ratios have also been reported in transition-zone tissue from larger prostates [34]. An aqueous ethanolic extract of Linderae Radix has also been reported to inhibit 5α-reductase activity in vitro [35]. These findings suggest that altered androgen metabolism may contribute to the inhibitory effects of LAE on TP-induced prostatic hyperplasia. However, circulating DHT concentrations depend on DHT formation and metabolic clearance [36], while the DHT/T ratio also depends on testosterone concentrations. Possible explanations for the reduced ratio include not only decreased testosterone-to-DHT conversion but also increased DHT clearance or higher testosterone concentrations resulting from reduced testosterone metabolism or clearance. In the present study, both control and LAE-treated rats were orchiectomized and received the same dose of TP. Differences in testicular testosterone production are unlikely to account for the reduced DHT/T ratio. Although the serum DHT/T ratio has been used as an indirect indicator of 5α-reductase activity [37], this activity was not directly measured in the present study. The reduced serum DHT/T ratio alone cannot establish direct inhibition of this enzyme by LAE.

Testosterone metabolism includes conversion to DHT by 5α-reductase and to estradiol by aromatase. To explore potential changes in local steroid metabolism, we examined 5α-reductase type 2 and aromatase protein expression in ventral prostate tissue by Western blotting. LAE treatment significantly reduced aromatase protein expression, whereas 5α-reductase type 2 expression did not differ significantly between the LAE and control groups. Local estrogen signaling has been implicated in BPH, with experimental evidence that estrogen signaling through G protein-coupled estrogen receptor promotes prostatic stromal cell proliferation and fibrosis [38]. The lower aromatase expression observed with LAE raises the possibility of altered local estrogen synthesis and signaling involved in prostatic growth and stromal remodeling. However, protein abundance alone cannot establish changes in enzyme activity or local estradiol concentrations.

An imbalance between cell proliferation and apoptosis has been implicated in the development of BPH [39]. Bax and Bcl-2 exert opposing effects on apoptotic susceptibility [40], while the caspase-9–caspase-3 cascade participates in the mitochondrial apoptotic pathway [41]. In human BPH tissue, finasteride treatment has been associated with epithelial atrophy and transient apoptosis [42], as well as activation of caspase-9 and caspase-3 [43]. A preoperative pilot study also reported an increased prostatic Bax/Bcl-2 ratio and changes suggestive of increased caspase-3 activity following treatment with a *Serenoa repens* extract [44]. In the present study, LAE significantly increased Bax and cleaved caspase-3 protein levels, as well as the cleaved caspase-3/procaspase-3 ratio, with the caspase-3 findings supporting enhanced activation of this key executioner of apoptosis. Although the cleaved caspase-9/procaspase-9 ratio increased, this change was accompanied by reduced procaspase-9 expression without a significant increase in cleaved caspase-9, limiting its interpretation as evidence of enhanced caspase-9 activation. Together, these findings suggest that modulation of apoptosis-related signaling may contribute to the attenuation of TP-induced prostatic hyperplasia by LAE.

Several constituents reported in the roots of *L. aggregata* may contribute to the effects observed in the present study. Germacrone, a sesquiterpenoid identified in root essential oil [45], inhibited 5α-reductase in vitro and attenuated testosterone-induced LNCaP cell proliferation and hamster flank-gland growth [46]. Zerumbone, reported as a major constituent of root essential oil [45], induced apoptosis in PC-3 and DU145 prostate cancer cells through mechanisms involving mitochondrial dysfunction and endoplasmic reticulum stress [47]. Boldine, a representative aporphine alkaloid found in the roots [48], also induced intrinsic apoptosis in DU145 cells, with an increased Bax/Bcl-2 ratio and caspase activation [49]. However, findings from prostate cancer cells cannot be directly extrapolated to benign prostatic tissue. These constituents were not identified or quantified in LAE, and their potential contributions, along with those of other constituents, remain to be established.

Previous studies have provided preliminary safety information for Linderae Radix. Linderae Radix showed low acute oral toxicity (LD_50_ > 10 g/kg in rats and mice) and negative results in Ames, mouse micronucleus, and sperm-abnormality assays [50]. In a 30-day oral toxicity study in rats, Linderae Radix at 5.0 g/kg produced no significant serum biochemical changes or treatment-related histopathological alterations, despite an increase in relative liver weight that the authors considered non-pathological [50]. In an 8-week randomized, placebo-controlled trial, a three-herb formulation containing Linderae Radix extract was well tolerated, with no serious adverse events or withdrawals due to adverse events [51]. However, differences in preparation and exposure conditions limit extrapolation to LAE. Further assessment of the safety of LAE is therefore warranted.

The present study supports the biological activity of LAE as a whole extract, but the contributions of individual constituents and the mechanisms underlying its effects remain to be established. Further quantitative characterization, fractionation, and evaluation of individual constituents would help identify the components contributing to the observed effects. Direct assessment of 5α-reductase and aromatase activities would help clarify whether LAE affects local conversion of testosterone to DHT and estradiol.

Taken together, LAE attenuated TP-induced prostatic hyperplasia in rats, as demonstrated by reduced prostate weights and indices and improved histopathological findings. The mechanistic implications of the accompanying changes in androgen-related measures and apoptosis-related proteins remain hypothesis-generating. These findings provide experimental evidence for a previously unexplored effect of Linderae Radix on prostatic hyperplasia and support further investigation of LAE as a potential therapeutic option for BPH.

## 4. Materials and Methods

### 4.1. Preparation of LAE

Dried roots of *L. aggregata* (batch No. K1152-PA1) were obtained from Gwangmyeongdang Pharmaceutical Co. (Ulsan, Korea). The material was authenticated by Prof. Jungbin Song (College of Korean Medicine, Kyung Hee University), and a voucher specimen (No. 24070304) was deposited in the herbarium of the same college. Dried roots (150 g) were extracted once with 1500 mL of 70% (*v*/*v*) aqueous ethanol for 3 h in a flask fitted with a reflux condenser, using a heating mantle set to 70 °C. The extract was filtered through Whatman qualitative filter paper (No. 2, 8 μm particle retention; Cytiva, Marlborough, MA, USA) to remove plant residues. The filtrate was concentrated at 45 °C using a rotary evaporator (N-1200A; EYELA, Tokyo, Japan), with the pressure gradually reduced from 120 hPa to 50 hPa. The concentrate was pre-frozen at −45 °C in a low-temperature bath (PFR-1000; EYELA) and subsequently lyophilized using a freeze dryer (FDU-1200; EYELA) at a pressure of approximately 15 Pa. The extraction yield was 12.05%, calculated as the mass of the lyophilized extract divided by the initial dry root mass × 100. LAE was stored at 4 °C until use.

### 4.2. HPLC Analysis of LAE

Norisoboldine was selected for the HPLC analysis of LAE because it is specified as a quantitative marker for Linderae Radix in the Chinese Pharmacopoeia (2025 edition) [52]. Quantification was performed using an Agilent 1260 Infinity III HPLC system (Agilent Technologies, Santa Clara, CA, USA) equipped with a G7111A quaternary pump, a G7129A vialsampler, and a G7115A diode-array detector. The norisoboldine reference standard (purity, 98.10%) was purchased from ChemFaces (Wuhan, China).

The reference standard (1 mg) was mixed with 1 mL of methanol/0.5% hydrochloric acid (2:1, *v*/*v*), and LAE was prepared using the same solvent. Both preparations were sonicated for 5 min. The LAE solution was filtered through a 0.2 μm polyvinylidene fluoride syringe filter before injection.

Separation was achieved on a ZORBAX Eclipse C18 column (4.6 × 250 mm, 5 μm; Agilent Technologies) at room temperature. Mobile phase A was water containing 0.1% formic acid and 0.1% triethylamine, and mobile phase B was acetonitrile. The gradient program was as follows: 0–13 min, 10–22% B; 13–22 min, 22% B; 22–25 min, 22–100% B; and 25–35 min, 100% B. The flow rate was 1.0 mL/min, with an injection volume of 10 μL and a detection wavelength of 280 nm. Chromatographic data were acquired and processed using OpenLab CDS software (version 3.7; Agilent Technologies). Norisoboldine was quantified by comparing sample peak areas with those obtained from reference standard solutions of known concentrations.

The HPLC method was validated for specificity, linearity, LOD, LOQ, accuracy, repeatability, and intermediate precision according to ICH guidelines [53].

### 4.3. Animals

Male Sprague–Dawley rats (10 weeks old) were purchased from Koatech (Pyeongtaek, Republic of Korea) and acclimatized for 1 week before experimentation. Animals were housed at 23 ± 1 °C and 40–60% relative humidity under a 12-h light/dark cycle. Food and water were provided ad libitum throughout the acclimation and experimental periods. All animal procedures were conducted in accordance with the Guidelines for the Care and Use of Laboratory Animals of Kyung Hee University. The experimental protocol was approved by the Institutional Animal Care and Use Committee of Kyung Hee University (approval No. KHSASP-24-170; approval date: 3 April 2024).

### 4.4. Induction of BPH and Treatment

After acclimatization, rats were randomly assigned to four groups based on body weight: sham (*n* = 5), control (*n* = 11), finasteride (1 mg/kg/day; positive control, *n* = 6), and LAE (200 mg/kg/day; *n* = 13). Group sizes were selected based on unpublished preliminary data from our laboratory and sample sizes reported in comparable 14-day TP-induced BPH studies [54,55], while considering the reduction principle of the 3Rs. Fewer animals were assigned to the sham and finasteride reference groups because preliminary data indicated relatively low within-group variability in the total prostate index, whereas more animals were allocated to the control and LAE groups because the LAE–control comparison was the principal efficacy comparison. No formal a priori sample-size calculation was performed. All randomized animals completed the experimental protocol and were included in the analyses.

All rats except those in the sham group underwent bilateral orchiectomy under isoflurane inhalation anesthesia using the abdominal approach described by Brown [56]. A midline abdominal incision was made, and both testes, epididymides, and epididymal fat were removed. Following tissue removal, the abdominal wall and skin were sutured. Sham-operated rats underwent incision and suturing without tissue removal. Animals were allowed to recover for 1 week before treatment.

Prostatic hyperplasia was induced in the control, finasteride, and LAE groups by daily subcutaneous administration of TP (3 mg/kg; TCI, Tokyo, Japan) in olive oil at an injection volume of 1 mL/kg for 14 days. The sham group received olive oil alone using the same route, volume, and schedule. During this period, LAE (200 mg/kg) or finasteride (1 mg/kg; PROSCAR^®^, Organon, Jersey City, NJ, USA) was dissolved in distilled water and administered orally once daily at a volume of 10 mL/kg. The sham and control groups received the same volume of distilled water orally on the same schedule. The finasteride dose was selected based on a previous study demonstrating its efficacy at 1 mg/kg in a rat model of TP-induced prostatic hyperplasia [57]. Finasteride was included as a pharmacological positive control to assess the model’s responsiveness to treatment and provide a reference for interpreting the effects of LAE.

### 4.5. Blood Collection and Prostate Dissection

After the 14-day dosing period, rats were fasted overnight and weighed the following day before sacrifice. Under isoflurane anesthesia, blood was collected from the inferior vena cava into serum separator tubes and kept on ice until centrifugation. Serum was separated by centrifugation at 3500 rpm for 10 min at 4 °C (5810R, Eppendorf, Hamburg, Germany) and stored at −80 °C until analysis.

The ventral, dorsolateral, and anterior prostate lobes were dissected, and the wet weight of each lobe was measured at the experimental endpoint. Ventral prostate tissue for histological analysis was fixed in 10% neutral-buffered formalin at 4 °C, while tissue for Western blot analysis was snap-frozen in liquid nitrogen and stored at −80 °C.

The prostate index was expressed as wet prostate weight (mg) per 100 g of fasted body weight. Inhibition rates for TP-induced increases in prostate weight and prostate index were calculated after correction for sham values using the following formula: Inhibition (%) = [(control value − treatment value)/(control value − sham value)] × 100.

### 4.6. Histological Analysis

Transverse sections (4 μm) were prepared from the central region of formalin-fixed ventral prostate tissues after dehydration and paraffin embedding. The sections were stained with H&E and examined using a light microscope (Eclipse Ci-L; Nikon, Tokyo, Japan).

Histological changes were assessed using a semiquantitative scoring system based on previously described methods [58,59]. The assessment included glandular architecture and epithelial, stromal, and basement membrane alterations, with higher scores indicating more severe pathological changes. For each animal, five randomly selected fields at 100× magnification and five at 400× magnification were examined. Two investigators blinded to treatment allocation independently scored the sections using the same predefined criteria for the severity and distribution of histological abnormalities. The total scores assigned by the two investigators were averaged to obtain a single histological score for each animal.

For each animal, five regions of the prostatic epithelium were randomly selected on images captured at 100× magnification. ImageJ software (version 1.54a; NIH, Bethesda, MD, USA) was used to measure the distance between the basement membrane and the luminal surface along a line perpendicular to the epithelial lining. The five measurements were averaged to obtain the mean epithelial thickness for each animal.

### 4.7. Measurement of Serum Testosterone and DHT Levels

Competitive enzyme-linked immunosorbent assay kits supplied by Cusabio (Wuhan, China) were used to quantify serum testosterone (CSB-E05100r) and DHT (CSB-E07879r). Both ELISA kits were validated by the manufacturer for sensitivity, precision, dilution linearity, and spike recovery. Diluted serum samples were analyzed following the manufacturer’s protocols. Absorbance was measured using an Epoch 2 microplate spectrophotometer (Agilent Technologies, Santa Clara, CA, USA), with detection and reference wavelengths set to 450 and 600 nm, respectively. For each assay, hormone concentrations were calculated from a standard curve generated using four-parameter logistic regression.

### 4.8. Western Blotting

Ventral prostate tissues were homogenized in RIPA lysis and extraction buffer (Thermo Scientific, Waltham, MA, USA) using a Bead Ruptor Elite homogenizer (OMNI International, Kennesaw, GA, USA). After centrifugation at 15,000× *g* for 20 min at 4 °C, protein concentrations in the supernatants were determined using a Pierce BCA Protein Assay Kit (Thermo Scientific). Samples were prepared with 4× Laemmli sample buffer (Bio-Rad Laboratories, Hercules, CA, USA).

For Bax and Bcl-2, 50 μg of protein per lane was separated by sodium dodecyl sulfate–polyacrylamide gel electrophoresis on 4–20% Mini-PROTEAN TGX precast gels (Bio-Rad Laboratories) at 110 V for 60–70 min. For caspase-9 and caspase-3, 10 μg of protein per lane was separated on 12% gels (110 V, 80 min) and 15% gels (100 V, 70 min), respectively. For 5α-reductase type 2 and aromatase, 10 and 30 μg of protein per lane, respectively, were separated on 12% gels at 110 V for 70 min. Proteins were transferred to polyvinylidene fluoride (PVDF) membranes using an iBlot 3 system and mini PVDF transfer stacks (IB34002; Invitrogen, Waltham, MA, USA) at 25 V for 3 min with medium cooling.

Membranes were blocked with 5% skim milk in Tris-buffered saline containing Tween 20 (TBST) at room temperature for 80–120 min and incubated with primary antibodies overnight at 4 °C. Antibodies against Bax (sc-7480; 1:200), Bcl-2 (sc-7382; 1:100), and β-actin (sc-47778; 1:1000) were obtained from Santa Cruz Biotechnology (Dallas, TX, USA). Antibodies against caspase-9 (9508; 1:1000) and caspase-3 (14220; 1:500) were obtained from Cell Signaling Technology (Danvers, MA, USA). Antibodies against 5α-reductase type 2 (MA5-37985; 1:1000) and aromatase (A12238; 1:1000) were obtained from Invitrogen and ABclonal (Woburn, MA, USA), respectively.

Secondary antibody incubation was performed at room temperature for 1 h using horseradish peroxidase-conjugated goat anti-mouse IgG (H + L) (31430; Invitrogen; 1:1000 for Bax, Bcl-2, and caspase-9; 1:10,000 for β-actin) or goat anti-rabbit IgG (H + L) cross-adsorbed antibody (G-21234; Thermo Fisher Scientific, Waltham, MA, USA; 1:5000 for caspase-3; 1:10,000 for 5α-reductase type 2; 1:1000 for aromatase). Each antibody incubation was followed by four washes with TBST for 10 min each.

Protein bands were visualized using NICSRO-WEST ECL Advance chemiluminescent substrates (Bionics, Seoul, Korea) and a ChemiDoc MP Imaging System (Bio-Rad Laboratories). Band intensities were quantified after background correction using Image Lab software (version 6.1; Bio-Rad Laboratories) and normalized to β-actin. The Bax/Bcl-2, cleaved caspase-9/procaspase-9, and cleaved caspase-3/procaspase-3 ratios were calculated from the quantified protein levels. Relative protein expression levels and protein ratios were expressed relative to the corresponding control-group mean, which was set to 1.

### 4.9. Statistical Analysis

Data are expressed as mean ± standard deviation (SD). Histological scores were analyzed using the Kruskal–Wallis test followed by Dunn’s multiple comparisons test. Other outcomes were analyzed using one-way analysis of variance (ANOVA) followed by Dunnett’s multiple comparisons test. Multiple comparisons were performed against the TP-treated control group. Following ARRIVE 2.0 recommendations, the standardized mean difference in total prostate index between the LAE 200 mg/kg and control groups was estimated as Hedges’ *g* with a two-sided 95% confidence interval [60]. Group-comparison tests were conducted using GraphPad Prism version 8.4.2 (GraphPad Software, San Diego, CA, USA). The effect-size calculation was performed in R version 4.6.1 (R Foundation for Statistical Computing, Vienna, Austria) with the effectsize package version 1.0.3 [61]. Differences were considered statistically significant at *p* < 0.05.

## Figures and Tables

**Table 1:** Histological scoring of the ventral prostate.

Histological Parameter	Sham	Con	Fina	LAE
**Low magnification (100×)**				
Luminal shape	1.67 (1.15)	2.18 (1.17)	2.00 (1.10)	2.23 (1.24)
Acinar shape	1.67 (1.15)	3.18 (1.40)	1.33 (0.82) *	1.77 (1.01) *
Interacinar space	1.00 (0.00)	3.18 (2.09)	1.00 (0.00) *	1.62 (1.50)
Stroma	1.00 (0.00)	2.82 (1.40)	1.00 (0.00) **	1.77 (1.01)
**High magnification (400×)**				
Epithelial shape	1.13 (0.23)	2.05 (0.76)	1.40 (0.36)	1.38 (0.27)
Number of epithelial layers	1.27 (0.46) **	2.45 (0.32)	1.60 (0.22) *	1.34 (0.39) ***
Layer distribution	0.00 (0.00) *	2.91 (1.14)	0.00 (0.00) ***	0.23 (0.83) ***
Polarity	2.33 (1.15)	2.82 (0.60)	2.00 (1.10)	1.62 (0.96) *
Epithelial piling	2.00 (1.73)	3.00 (0.00)	2.00 (1.55)	1.15 (1.52) **
Budding into stroma	3.33 (2.89)	4.09 (2.02)	0.00 (0.00) **	2.31 (2.59)
Periacinar epithelial clusters	3.00 (0.00)	1.91 (1.51)	1.50 (1.64)	1.62 (1.56)
Isolated epithelial clusters	0.00 (0.00)	2.27 (2.61)	0.00 (0.00) *	0.00 (0.00) **
Lesion distribution	1.33 (1.15)	5.27 (2.41)	3.67 (2.94)	2.31 (2.14) *
Nuclear shape	1.00 (0.00)	3.91 (1.87)	2.33 (2.07)	2.23 (1.92)
Nuclear size	2.00 (0.00)	3.27 (1.01)	2.33 (0.82)	2.62 (0.96)
Mitotic figures	0.67 (1.15)	2.27 (1.95)	0.67 (1.03)	0.46 (0.88) *
Basement membrane integrity	1.00 (0.00)	2.45 (2.02)	1.67 (1.63)	1.31 (1.11)
Basement membrane thickness	1.00 (0.00)	3.55 (2.02)	1.67 (1.63)	1.31 (1.11) **
**Total score**	25.40 (3.99) *	53.60 (10.84)	26.17 (6.55) **	27.26 (8.92) ***

* *p* < 0.05, ** *p* < 0.01, and *** *p* < 0.001 vs. control (Con). Fina, finasteride 1 mg/kg; LAE, *Lindera aggregata* root extract 200 mg/kg. Values are mean (SD).

## Data Availability

The datasets generated and analyzed during the current study are available from the corresponding author upon reasonable request.

## Abbreviations

The following abbreviations are used in this manuscript:

3Rs	Replacement, reduction, and refinement
5-ARIs	5α-Reductase inhibitors
ANOVA	Analysis of variance
AP	Anterior prostate
ARRIVE	Animal Research: Reporting of In Vivo Experiments
Bax	Bcl-2-associated X protein
Bcl-2	B-cell lymphoma 2
BPH	Benign prostatic hyperplasia
DHT	Dihydrotestosterone
DLP	Dorsolateral prostate
ELISA	Enzyme-linked immunosorbent assay
H&E	Hematoxylin and eosin
HPLC	High-performance liquid chromatography
ICH	International Council for Harmonisation of Technical Requirements for Pharmaceuticals for Human Use
LAE	*Lindera aggregata* root extract
LD_50_	Median lethal dose
LOD	Limit of detection
LOQ	Limit of quantification
LUTS	Lower urinary tract symptoms
PVDF	Polyvinylidene fluoride
RSD	Relative standard deviation
SD	Standard deviation
T	Testosterone
TBST	Tris-buffered saline containing Tween 20
TP	Testosterone propionate
VP	Ventral prostate
